# Methemoglobinemia in Patient with G6PD Deficiency and SARS-CoV-2 Infection

**DOI:** 10.3201/eid2609.202353

**Published:** 2020-09

**Authors:** Kieran Palmer, Jonathan Dick, Winifred French, Lajos Floro, Martin Ford

**Affiliations:** King’s College Hospital National Health Service Foundation Trust, London, UK

**Keywords:** severe acute respiratory syndrome coronavirus 2, SARS CoV-2, coronavirus, viruses, coronavirus disease, COVID-19, hemolytic anemia, methemoglobinemia, glucose-6-phosphate dehydrogenase, G6PD, deficiency, respiratory infections, zoonoses

## Abstract

We report a case of intravascular hemolysis and methemoglobinemia, precipitated by severe acute respiratory syndrome coronavirus 2 infection, in a patient with undiagnosed glucose-6-phosphate dehydrogenase deficiency. Clinicians should be aware of this complication of coronavirus disease as a cause of error in pulse oximetry and a potential risk for drug-induced hemolysis.

Coronavirus disease is a novel infectious disease that primarily manifests as an acute respiratory syndrome but can also cause multiorgan dysfunction. Severe acute respiratory syndrome coronavirus 2 (SARS-CoV-2) infection has been documented to cause vasoocclusive crisis and acute chest syndrome in patients with sickle cell anemia (*1*). We report another potentially major complication of infection in a patient with a common enzymatic disorder.

Glucose 6‐phosphate dehydrogenase (G6DP) deficiency is an X-linked enzymatic disorder that affects 400 million persons worldwide and has a high prevalence (5%–20%) in African and Asian populations (*2*). G6DP catalyzes the formation of nicotinamide adenine dinucleotide phosphate (NADPH) (*3*). NADPH maintains hemoglobin in the ferrous state by forming reduced glutathione, which prevents oxidative damage (*3*). G6DP deficiency increases the risk for intravascular hemolysis upon exposure to oxidative agents, such as fava beans, sulfonamides, and hydroxychloroquine, the subject of clinical trials for persons with SARS-CoV-2 infection.

G6PD deficiency can induce methemoglobinemia by inhibiting NADPH-flavine reductase, which prevents the reduction of methemoglobin. Methemoglobin is unable to bind to oxygen, and the remaining oxyhemoglobin develops heightened oxygen affinity and diminished delivery, leading to tissue hypoxia (*4*). Viral infections, including HIV, hepatitis viruses (A, B, and E), and cytomegalovirus, can precipitate intravascular hemolysis in patients with G6PD deficiency (*5**,**6*). Concurrent methemoglobinemia has also been reported in the context of viral-induced hemolysis (*5*).

A 62-year-old Afro-Caribbean man with a medical history of type 2 diabetes and hypertension came to the hospital for a 5-day history of fever, dyspnea, vomiting, and diarrhea. Auscultation of his chest showed bilateral crackles. He was tachycardic, hypotensive, and dehydrated, with a prolonged capillary refill time and dry mucous membranes.

Laboratory tests showed an acute kidney injury. Blood urea nitrogen was 140 mg/dL, creatinine 5.9 mg/dL (baseline 1.1 mg/dL), capillary blood glucose >31 mmol/L, and blood ketones 1.1 mmol/L. A chest radiograph showed bilateral infiltrates, and a result for a SARS-CoV-2 reverse transcription PCR specific for the RNA-dependent RNA polymerase gene was positive (validated by Public Health England, London, UK).

The patient was treated for SARS-CoV-2 pneumonitis and a hyperosmolar hyperglycemic state with crystalloid fluid, oxygen therapy, and an insulin infusion. His creatinine increased to 9.3 mg/dL, suspected secondary to hypovolemia and viremia, and acute hemodialysis was started. Results of a screen for other causes of acute kidney injury, including renal ultrasonography and autoimmune serologic analysis, was unremarkable.

On day 7 postadmission, his peripheral oxygen saturations decreased, and oxygen therapy was increased to 15 L/min by use of a nonrebreather mask to maintain saturations of 80%. Arterial blood gas analysis revealed a partial pressure of oxygen of 22 kPa and an oxygen saturation of 100%. Co-oximetry showed a methemoglobin level of 6.5%. Repeat laboratory tests showed hemolytic anemia; hemoglobin was 52 g/L, haptoglobin <0.1 mg/dL, and lactate dehydrogenase 1,566 U/L. A direct antiglobulin test excluded major immune-mediated hemolysis. A blood film for the patient showed normochromic normocytic erythrocytes and a few hemighost cells (Figure, panel A). A 2-stage G6DP assay confirmed G6DP deficiency (0.8 IU/g hemoglobin).

**Figure:**
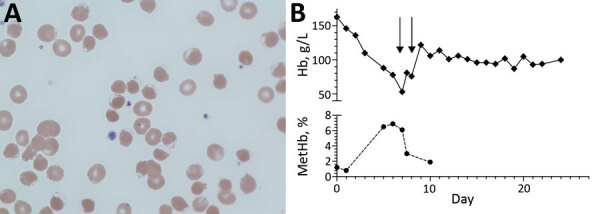
Testing of patient with G6PD deficiency and SARS-CoV-2 infection, United Kingdom. A) Blood film showing normochromic normocytic erythrocytes and a few hemighost cells. Hemighost cells are formed after oxidative hemolysis seen in G6DP deficiency. Hb is contracted to 1 pole of the cell, leaving an unfilled area enclosed by an intact membrane (original magnification ×100). B) Hb and metHb concentration during admission. Each arrow indicates a 3-unit erythrocyte transfusion. G6PD, glucose-6-phosphate dehydrogenase; Hb, hemoglobin; metHb, methemoglobin; SARS-CoV-2, severe acute respiratory syndrome coronavirus 2.

The patient was given 2 blood transfusions (Figure, panel B) and oxygen therapy. His medication history included amoxicillin/clavulanic acid, heparin, amlodipine, and metformin, which did not indicate a precipitant for the hemolytic crisis. The methemoglobinemia gradually resolved, and his oxygen requirements decreased. He recovered dialysis-independent renal function. He was given folic acid (5 mg/d) and discharged 22 days after admission.

The mechanism by which SARS-CoV-2 causes hemolysis is unknown. Other viral infections have been reported to produce reactive oxygen and nitrogen species, which impair intracellular proteins and DNA in cells with damaged antioxidant enzyme metabolism (*7*). The concurrent secondary methemoglobinemia in this case also suggests oxidative stress and impaired redox balance.

Patients with G6DP deficiency might be more vulnerable to SARS-CoV-2 infection (*8*). Infection of G6DP-deficient lung cells with human coronavirus 229E resulted in increased viral production and replication compared with normal cells (*9*). An increased susceptibility to infection and hemolysis with secondary tissue hypoxia might result in increased illness and death (*8*).

Hydroxychloroquine has been proposed as a treatment for SARS-CoV-2 infection and is considered safe in usual therapeutic doses in class II or III G6PD deficiency. However, caution is advised with higher doses because data for this setting are limited. Oxidative stress might contribute to the pathogenesis of severe SARS-CoV-2 infection (*10*). Evaluation of parameters of oxidative stress in SARS-CoV-2 are currently underway (ClinicalTrials.gov identifier NCT04375137) and might determine whether there is an increased risk for drug-induced hemolysis in patients with G6PD deficiency.

Treatment for methemoglobinemia with intravenous methylene blue is recommended if the blood methemoglobin level is >20%–30%. However, in G6DP deficiency, treatment with methylene blue is contraindicated because the reduction of methemoglobin is NADPH dependent. This finding might precipitate intravascular hemolysis and therapy with ascorbic acid or supportive treatment with oxygen as indicated instead. During the SARS-CoV-2 pandemic, clinicians must be aware of the possible increased susceptibility of patients with G6DP deficiency to severe hemolytic crises and the consequences for investigation and treatment.
